# Anthocyanin-Binding Affinity and Non-Covalent Interactions with IIS-Pathway-Related Protein Through Molecular Docking

**DOI:** 10.3390/cimb47020087

**Published:** 2025-01-29

**Authors:** Haroon, Zahid Khan, Wasim Javaid, Lian-Xi Xing

**Affiliations:** 1 College of Forestry and Landscape Architecture, South China Agricultural University, Guangzhou 510642, China; 2 College of Life Sciences, Northwest University, Xi’an 710069, China; khanzahid370@yahoo.com

**Keywords:** polyphenols, aging, insulin signaling pathway, in silico analysis

## Abstract

Anthocyanins compounds, including cyanidin, malvidin, pelargonidin, peonidin, and petunidin, have demonstrated remarkable anti-aging and insulin-sensitizing properties through their interactions with proteins associated with the insulin/insulin-like growth factor signaling (IIS) pathway in *Reticulitermes chinensis*, employing advanced molecular docking techniques to elucidate strong binding affinities between specific anthocyanins and key proteins such as *Pdk1*, *EIF4E*, and *Tsc2* in *R. chinensis*, suggesting a potential mechanism for their anti-aging effects. These findings not only provide critical insights into the therapeutic potential of anthocyanins for mitigating insulin resistance and promoting longevity, but also highlight the efficacy of in silico molecular docking as a predictive tool for small-molecule–protein interactions. Our research opens new avenues for the development of innovative therapeutic strategies targeting age-related diseases. However, further investigations, including a comprehensive chromosomal analysis and in vivo studies, are essential in order to fully elucidate the molecular mechanism underlying these interactions and their physiological implications. The detailed characterization of anthocyanin-binding affinities and their interactions with key regulatory genes presents exciting opportunities for advancement in molecular medicine, pharmacology, and the development of novel nutraceuticals.

## 1. Introduction

Physiological, metabolic, and reproductive alterations are key features of the aging process in all organisms [1,2,3,4]. Age-related disorders, including neurological diseases, immune system dysfunction, and cancer, are often associated with dysfunction in proteolytic systems, leading to the accumulation of damaged intracellular proteins and a shortened lifespan [3,5,6,7,8,9]. Several genes and proteins play a critical role in regulating longevity in model organisms associated with the insulin/IGF-1 signaling (IIS) pathway, which modulates the response to environmental stimuli and controls metabolic activity [10,11,12,13]. Mutations in these genes can influence the evolutionary expression of aging-related genes, supporting the classical evolutionary theory of aging, which has been supported by extensive research [10,14,15]. In the early 1990s, *Caenorhabditis elegans* was presented as a model organism for aging research [2,16]. Since then, a variety of model organisms, from yeast to mammals, have been used to investigate the mechanisms underlying aging and longevity [17,18,19,20]. Mammalian models, particularly rodents and non-human primates, offer valuable insights into aging processes due to their close evolutionary relationship with humans [16,21,22,23]. These models provide relevant information for understanding human aging mechanisms. However, they come with significant drawbacks, including long lifespans, substantial space and resource requirements, and high costs associated with breeding and maintenance [22,24]. In contrast, alternative model organisms present several advantages for aging research. These include fruit flies (*Drosophila melanogaster*), silkworms, grasshoppers, and other social insects such as bees, ants, and termites. These organisms are characterized by shorter lifespans, lower maintenance costs, and simpler breeding requirements [16,21,25]. These features make them attractive alternatives for studying aging processes, potentially allowing for more rapid and cost-effective research outcomes.

Over the past two decades, extensive research has focused on several age-related pathways, including the mitogen-activated protein kinase (MAPK), AMP-activated protein kinase (AMPK), and insulin signaling pathways [26,27,28,29,30]. Key enzymes involved in the IIS and AMPK pathways, such as sirtuin-3 (SIRT3), sirtuin-6 (SIRT6), and 5′-AMP-activated protein kinase catalytic subunit alpha-2 (AMPK2), play pivotal roles in regulating oxidative stress and cellular energy homeostasis, particularly during conditions like caloric restriction [31,32]. Within the IIS pathway, three proteins, insulin-like growth factor 1 receptor (IGF1R), phosphoinositide-dependent kinase-1 (PDK-1), and rac-alpha serine/threonine-specific kinase 1 (Akt1), have demonstrated significant influence on aging and longevity across a diverse range of organisms [33,34].

Numerous studies have demonstrated the associations of crude anthocyanin extracts to various aging related pathways in model organisms, including *C. elegans*, *D. melanogaster*, and mice [35,36,37]. However, the pure anthocyanins most commonly investigated for their biological effects are cyanidin, delphinidin, and peonidin [38,39,40]. The complete spectrum of biological functions of these pure anthocyanins remains to be fully elucidated [40,41,42]. Anthocyanins, a subclass of flavonoid compounds, are categorized into several types based on their chemical structure. The six major anthocyanins comprise cyanidin, delphinidin, malvidin, pelargonidin, and peonidin, each characterized by similar aromatic rings but distinct side chains [40,42]. Among these, delphinidin possesses the highest number of hydroxyl groups (six), followed by cyanidin and petunidin (five), and anthocyanins have four hydroxyl groups. The quantity and position of these hydroxyl groups may significantly influence their antioxidant properties and biological activity [43,44].

This study aimed to investigate the potential mechanism of action of pure anthocyanin compounds in aging using molecular docking techniques. The primary objectives were to screen anthocyanin compounds for lead-likeness, rank their binding affinity with various aging-related enzymes, and dock top-binding anthocyanin compounds with crystal structures of aging-related proteins associated with the IIS pathway of *R. chinensis*. The purpose of this research was to formulate evidence-based hypotheses regarding the potential mechanism through which anthocyanins exert their effects on aging in their natural state, contributing to our understanding of anti-aging processes. Researchers screened these compounds for lead-likeness and ranked their binding affinity with various aging-related enzymes [40]. Based on these analyses, evidence-based hypotheses have been formulated regarding the potential mechanism through which anthocyanins exert their effects on aging in their natural state.

## 2. Materials and Methods

### 2.1. Termite Collection

*Reticulitermes chinensis* colonies were established from alates collected in Chengdu, China and reared as founder colonies with one male and female (alate) per container at 25 °C in the termite lab at Northwest University, Xian, 710069, China. The initial colonies were built from pine sawdust, which was rich in anthocyanin [45]. Anthocyanins, beyond their known physiological benefits in plants, are proposed to play diverse roles in plant–animal interactions, including attracting pollinators and frugivores while repelling herbivores and parasites [46]. After four years of rearing, individuals were classified as secondary worker reproductive king (SWRK) and queen (SWRQ), primary kings (PKs), primary queens (PQs), and workers (male “WM”s and female “WF”s), and then preserved in liquid nitrogen at −80 °C. Gender differentiation of WM, WF, PQ, PK, SWRK, and SWRQ was performed through microscopic examination of the seventh sterna. No special permits were required for sampling and experimentation as *R. chinensis* is not endangered or protected.

### 2.2. RNA Extraction and Illumina Sequencing

Three replicates from each caste (WM, WF, PK, PQ, SWRK, and SWRQ) were pooled to obtain sufficient RNA for cDNA library construction. Total RNA was extracted using TRIzol reagent and assessed with an Agilent 2100 Bioanalyzer (Agilent Technologies, Palo Alto, CA, USA). The NEBNext Ultra RNA Library Prep Kit for Illumina created cDNA libraries, starting with mRNA enrichment using Oligo(dT) beads, followed by fragmentation and reverse-transcription into cDNA. Second-strand cDNA synthesis involved DNA polymerase I, RNase H, dNTPs, and buffer. Purified cDNA fragments were end-repaired, poly(A)-tailed, and ligated to Illumina adapters. Ligation products were size-selected via agarose gel electrophoresis and PCR-amplified. Libraries were sequenced on the Illumina HiSeqTM 4000 platform by Gene Denovo Biotechnology Co. (Guangzhou, China).

### 2.3. Protein Sequences of Reticulitermes chinensis Castes

Phosphoinositide-dependent kinase-1 (Pdk1), eukaryotic translation initiation factor 4E (EIF4E), and tuberous sclerosis complex 2 (TSC2) protein sequences were retrieved from the transcriptomic data of *R. chinensis* castes. This foundational dataset enables comprehensive analyses of protein structure, function, and evolutionary relationships across different castes. To further investigate the structural characteristics of these proteins, the SWISS-MODEL tool was employed to predict their structure based on the obtained sequences. These predicted models can then be compared with existing entries in the protein data bank (PDB) to validate and refine the structures, potentially offering novel insights into the structural biology of these proteins in *R. chinensis* and their roles in caste differentiation [47,48,49]. Various anthocyanins, including cyanidin (CID: 128861), delphinidin (CID: 68245), malvidin (CID: 159287), pelargonidin (CID: 440832), peonidin (CID: 441773), and petunidin (CID: 441774), were obtained from PubChem (www.pubchem.ncbi.nlm.nih.gov) [40]. Known inhibitory ligands for these proteins were identified through the PDB, and their corresponding data were subsequently acquired from PubChem.

### 2.4. Molecular Docking and Molecular Dynamics (MD) Simulation

The target proteins were meticulously prepared for molecular docking by downloading and processing them according to established protocols. This preparation involved adding hydrogen atoms and Gasteiger charges, followed by combining charges and removing non-polar hydrogen, lone pairs, water molecules, and non-standard residues, as outlined by Mcule (Mcule Inc., Palo Alto, CA, USA) and AutoDock Tools [50,51]. The compounds Pdk1, EIF4E, and Tsc2 were then docked to their respective target proteins using a standardized docking procedure (using AutoDock Vina with a grid box size of 60 × 60 × 60 Å and exhaustiveness of 8”). Molecular docking techniques were employed to predict the interaction energies and orientations of various anthocyanin inhibitors with the targeted proteins, allowing for a comprehensive analysis of potential binding modes. The docking simulations were conducted with a maximum of 5 conformations per ligand and a root mean square deviation (RMSD) tolerance of 2.0 Å [52,53]. To assess the strength of ligand–receptor interactions, the most negative docking score was utilized as a measure of the predicted interaction energy, with a cutoff value of −6.0 kcal/mol considered significant for further analysis. This approach enabled the identification of promising anthocyanin inhibitors with potential therapeutic applications while acknowledging the limitations of computational docking methods, such as the absence of protein flexibility and solvent effects in the simulations.

Molecular dynamics (MD) simulations were conducted on the HOLO form using NAMD software (Version 3.0.1) in conjunction with MOE to investigate the structural and dynamic properties of the protein and ligands [54]. The simulation parameters were carefully selected to ensure an accurate representation of the molecular interactions. The Amber10 force field, known for its reliability in biomolecular simulations, was employed along with the extended Hückel theory (EHT) to describe the electronic structure of the ligand molecules [54,55]. A reaction field (R-Field) dielectric constant of 1:80 was applied to account for long-range electrostatic interactions. A cutoff range of 8–10 Å was implemented for non-bonded interactions to balance computational efficiency and accuracy. The protein–ligand complexes obtained from docking simulation were solvated in water with appropriate ionic strength (0.2 mol/L NaCl) to mimic physiological conditions, and a margin of 12.0 Å was maintained to prevent artificial interactions between periodic images. Post-simulation trajectory analyses were conducted using visual molecular dynamics (VMD) software, which was employed to calculate key metrics such as root mean square deviation (RMSD) over a simulation time of 100 ns (nanoseconds) [56]. It is important to note that, while this simulation provides valuable insights, limitations exist due to the relatively short simulation time of 100 ns, which may not capture long-timescale conformational changes.

### 2.5. Physicochemical Analysis of Anthocyanin Compounds for Lead-like Properties

Data on the physicochemical properties of anthocyanin compounds and known inhibitory ligands were obtained from several databases, including PubChem, Mcule, ChemAxon, and Chemmine Tools. In accordance with Lipinski’s rule of five (Ro5) [57], several factors were considered in order to assess the lead-like potential of the ligands. These factors include molecular mass (≤500 Da), octanol/water partition coefficient (logP ≤ 5), hydrogen bond acceptors (≤10), and hydrogen bond donors (≤5). Additionally, we evaluated the topological polar surface area (TPSA ≤ 140 Å²) and the number of rotatable bonds (≤10) as extended measures of drug-likeness [58]. The anthocyanin compounds were analyzed against these criteria to determine their potential as lead-like molecules. Compounds meeting all or most of these criteria were considered to possess favorable drug-like properties, suggesting a higher likelihood of oral bioavailability and potential for further development as therapeutic agents [59]. It is important to note, however, that, while these rules provide useful guideline, exceptions exist among known drugs.

### 2.6. The Top-Binding Ligands and the Inhibitory Ligand Visualization of the Aging-Related Genes

PLIP (Protein–Ligand Interaction Profiler; Michael Schroeder group at the Biotechnology Center TU Dresden (BIOTEC), Dresden, Germany) was employed to analyze and compare the interactions between the top-binding anthocyanins and the amino acid residues of the target proteins [60,61]. This analysis focused on characterizing the number of amino acid residues involved and the non-covalent interactions between the ligands and receptors, in order to evaluate the reproducibility and reliability of the docking experiments. The non-covalent interactions examined included hydrogen bonds (with a distance cutoff of 3.5 Å), hydrophobic interactions (with a distance cutoff of 3.9 Å), π-stacking (with a distance cutoff of 5.5 Å), and salt bridges (with a distance cutoff of 5.5 Å) [59,62]. To validate the docking results, the generated docked poses of the protein–ligand complexes were superimposed onto the experimentally determined structure from the PDB. This superimposition was carried out using molecular dynamics (MD) simulation techniques and visual molecular dynamics (VMD) software (version VMD 1.9.4) (Theoretical and Computational Biophysics Group, University of Illinois at Urba-na-Champaign, South Wright Street Champaign, IL 61820, USA) for visualization and further analysis [63,64]. The ligand–protein interactions analysis provided valuable insights into the binding modes and affinities of the anthocyanins for aging-related protein products. The number of hydrogen bonds, hydrophobic interactions, and other non-covalent interactions were quantified and compared across different ligands to identify the most promising candidates for potentially inhibiting aging-related processes. Additionally, superimposition of the docked poses with those inhibitors enabled the evaluation of the docking protocol’s accuracy and identification of key structural features that contribute to binding affinity and specificity.

### 2.7. Quantitative Real-Time PCR (RT-qPCR)

In this study, primer pairs for genes associated with the insulin signaling pathway were designed using Primer3 v1.1.4 (Table 1). Total RNA was extracted from the entire bodies of individuals representing various castes (SWRK, SWRQ, PQ, PK, WF, and WM workers) utilizing the RNAsimple Total RNA Kit (Tiangen). The quality and quantity of the extracted RNA were assessed using a NanoReady spectrophotometer (Model: F-1100, China) to ensure purity with respect to protein and salt contamination. A cDNA library was constructed using the FastKing RT Kit (Tiangen), and the resulting cDNA was stored at −20 °C for subsequent experiments. Real-time quantitative PCR (RT-qPCR) was performed to amplify the cDNA using a CFX 96 instrument (Bio-Rad) with SuperReal PreMix Plus (Tiangen), following the manufacturer’s protocol. Beta-actin (RsACT) [65,66], previously evaluated as the most reliable reference gene for *Reticulitermes* termites [67], was utilized as an internal control. Relative gene expression was calculated using the standard 2^−∆∆Ct^ method [68]. To ensure robustness and reproducibility of the results, three biological and three experimental replicates were performed for each RT-qPCR experiments, with each replicate consisting of five individual samples.

## 3. Results and Discussion

### 3.1. Different Anthocyanin Compounds and Known Inhibitory Ligands Are Compared for Their Lead-like Qualities

The findings for each of the six types of anthocyanins, compared to a lead compound, are presented in Table S1. Except for delphinidin, none of the anthocyanin compounds violate Lipinski’s rule of five (Ro5) criteria. According to Ro5, poor absorption or permeation is more likely when there are more than five hydrogen bond donors, more than ten hydrogen bond acceptors, a molecular weight greater than 500 Da, or a calculated Log P (CLog P) greater than 5. Delphinidin violates the Ro5 by having more than five hydrogen bond donors [40]. Additionally, the amount of lead in each anthocyanin component was above the acceptable threshold, although one violation was below the limit of concern.

### 3.2. Biochemical and Structural Analysis

The biochemical and structural properties of Pdk1, Tsc2, and EIF4E were analyzed using the Expasy Server. EIF4E exhibited the highest amino acid count (296) and molecular weight (33,978.5 Da), closely followed by Pdk1, while Tsc2 displayed significantly lower values. A theoretical isoelectric point (pI) analysis revealed Tsc2 (10.77) to be the most basic protein, followed by Pdk1 (9.58) and EIF4E (8.93). EIF4E demonstrated the highest charge density, with the greatest number of both negatively and positively charged density, with the greatest number of both negatively charged residues. An elemental composition analysis showed similar proportions of atoms in EIF4E and Pdk1, while Tsc2 contained fewer atoms due to its smaller size. Pdk1 exhibited the highest extinction coefficient when cysteines were oxidized. Instability indices suggested Pdk1 as the most stable protein, while Tsc2 appeared less stable. EIF4E displayed the highest aliphatic index, indicating superior thermostability. The grand average of hydropathy (GRAVY) scores revealed Pdk1 and EIF4E to be more hydrophobic compared to the hydrophilic Tsc2. These findings elucidate the distinct chemical compositions and stability profiles of the three proteins, which may influence their functional roles and structural integrity (Table 2).

The comparative analysis of Pdk1, Tsc2, and EIF4E proteins reveals distinct stability and aggregation characteristics. Pdk1 demonstrates superior stability, with a hydrophobic fitness score of −17, an isoelectric point (pI) of 8, and the lowest total energy in both BUDE (−4272) and EvoEF2 (−1227) force field results. EIF4E exhibits moderate stability, with a lower pI of 5 and a total energy of −276 in the Rosetta results but shows a higher aggregation tendency (total aggregation score: −227). In contrast, Tsc2 displays the least stability among the three proteins, with a hydrophobic fitness of −15, a pI of 8, and the highest DFIRE2 total energy (−343), coupled with a lower aggregation score (−103). These findings underscore the diverse biophysical properties of these proteins, with Pdk1 emerging as the most stable, EIF4E showing a balance between stability and aggregation potential, and Tsc2 exhibiting relatively lower stability across the evaluated metrics (Table 3).

### 3.3. Docking of Several Anthocyanin Compounds In Silico to Proteins Associated with Aging

In silico docking was performed on various anthocyanin classes to identify potential interactions with genes involved in the aging process. The level of interaction between the ligand (anthocyanin) and target protein was quantified by the binding energy (kcal/mol), as shown in Table S2. The binding energies of the different anthocyanin compounds for genes linked to MAPK pathways, such as MAPKAPK2, MAPK10, and B-raf, were lower than those of recognized inhibitors, including PDY, ANP, and 215, respectively. This trend was consistent across all the protein analyzed. Each protein had a unique top-binding anthocyanin: for example, delphinidin was the top-binding one for MAPKAPK2, while pelargonidin and peonidin were the top-binding ones for MAPK10, and peonidin was the top-binding binder for B-raf. The binding energies of the anthocyanin compounds in the AMPK pathway were comparable to those of AR6 and TAK, which are inhibitors of sirt3 and AMPK2, respectively. In the case of MAPK10, the binding energy of the anthocyanins was significantly lower than that of the well-known inhibitor AR6. Notably, three anthocyanins, delphinidin, petunidin, and peonidin, were found to bind AMPK2, SIRT3, and SIRT6.

In the insulin signaling pathway, the binding energies of the IGF1R and PDK-1 inhibitors ANP and STU were higher than those of anthocyanin compounds. However, XFE, an inhibitor of Akt1 protein, showed binding energies comparable to those of the anthocyanin. Different proteins exhibited unique top-binding anthocyanin compounds: delphinidin was the top binder for IGF1R protein, while malvidin and petunidin were the top binders for PDK-1 protein. Similarly, malvidin and petunidin were the top binders for Akt1 protein.

### 3.4. Root Mean Square Deviation (RMSD)

**Figure 1:**
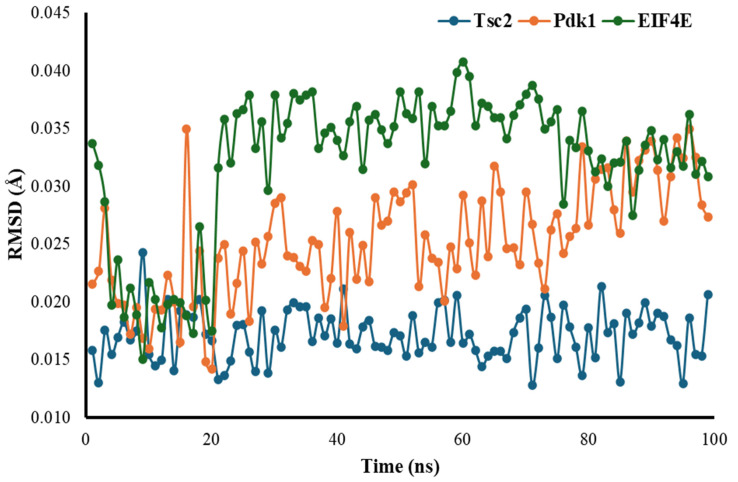
Calculated RMSD of Tsc2, Pdk1, and EIF4E proteins.

The RMSD for Tsc2 shows a relatively stable trend over the nanosecond timescale, fluctuating within a narrow range (approximately 0.015 to 0.020 Å) with minor variations, indicating overall stability without significant spikes, and remaining close to its baseline throughout. In contrast, the RMSD for Pdk1 exhibits greater variability, with initial fluctuations and peaks rising above 0.030 Å, particularly in the latter half of the simulation, suggesting that Pdk1 undergoes more dynamic conformational changes with a wider range of deviations. EIF4E, on the other hand, shows the highest variability, with more frequent spikes and fluctuations in structural stability. RMSD values for EIF2E often exceed 0.035 Å, with occasional peaks surpassing 0.040 Å, indicating more significant structural changes compared to Tsc2 and Pdk1 (Figure 1).

### 3.5. Evaluation of the Interactions Between the Top-Binding Binders of Anthocyanin

The non-covalent interactions between the top-binding anthocyanin compounds and the interacting amino acid residues were analyzed and compared with those of a known protein inhibitor. In order to verify the docking procedure, the inhibitory ligands for the protein of interest were re-docked and compared with the initial structure. All inhibitory genes had an overall root mean square deviation (RMSD) value of less than 1.2 angstroms (Å), indicating that the crystal structures of the re-docked ligand protein were comparable to the original ones. The results suggest that the molecular docking experiments provided accurate binding affinities predictions.

**Figure 2:**
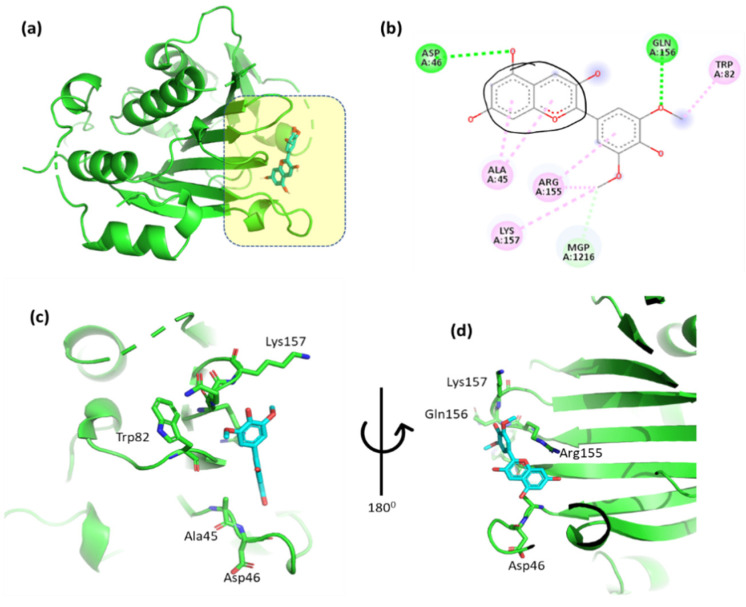
(**a**) Molecular docking analysis showing cyanidin to the active site of EIF4E protein. (**b**) Key interaction and visualization between protein and ligand active site residues, with colored dotted lines representing various types bonds. A close-up view (**c**,**d**) of interacting residue of EIF4E protein with ligand.

The docking results of cyanidin with EIF4E are highlighted in Figure 2 and Table S3, providing critical insight into the molecular interactions. These figures illustrate the binding pose of cyanidin and its key interaction with the EIF4E. Specifically, the 2,6-dimethoxyphenol moiety of cyanidin forms a hydrogen bond with Gln156 and a hydrophobic interaction with Trp82. Additionally, Ag155 exhibits an arene–arene (π–π stacking) interaction with the aromatic ring of the 2,6-dimethoxyphenol moiety, while the methoxy group interacts hydrophobically with Lys157. The double aromatic ring of cyanidin also forms hydrogen bonds with Asp46 and interacts hydrophobically with Ala45. Figure 2c,d provide close-up views of the residues involved in these interactions, offering a detailed understanding of the molecular dynamics. The inclusion of these figures is essential in order to visually support the detailed description of interactions and ensure clarity in interpreting the results.

**Figure 3:**
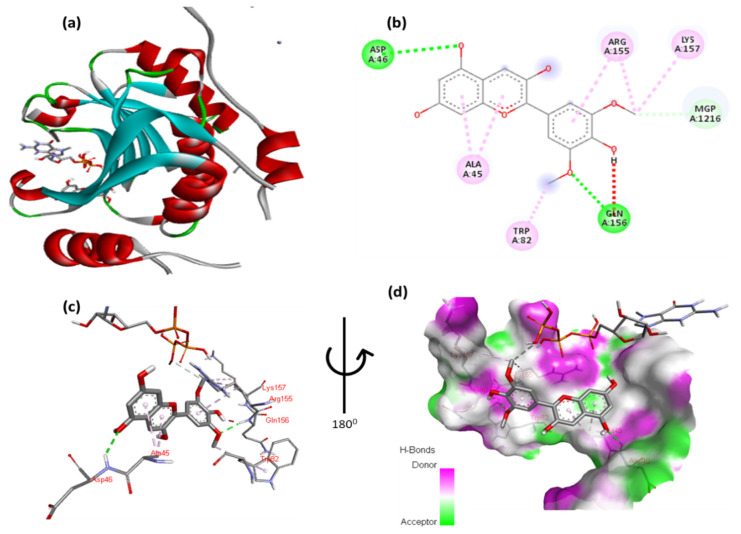
(**a**) Molecular docking analysis showing malvidin to the active site of EIF4E protein. (**b**) Key interaction and visualization between protein and ligand active site residues, with colored dotted lines representing various types bonds. A close-up view (**c**,**d**) of interacting residue of EIF4E protein with ligand.

The docking results of malvidin with EIF4E reveals an intricate molecular interaction, as illustrated in Figure 3 and Table S4. The 2,6-dimethoxyphenol moiety of malvidin engages in multiple interactions: it forms a hydrogen bond with Gln156 and a hydrophobic interaction with Trp82. Additionally, Ag155 exhibits one arene–arene (π–π stacking) interaction with the aromatic ring of the 2,6-dimethoxyphenol moiety, while its methoxy group forms a hydrophobic interaction with Lys157. The double aromatic ring of malvidin also forms hydrogen bonds with Asp46 and has a hydrophobic interaction with Ala45. Figure 3c,d provide detailed close-up views of these residue–ligand interactions, offering a comprehensive visualization of the binding mode.

**Figure 4:**
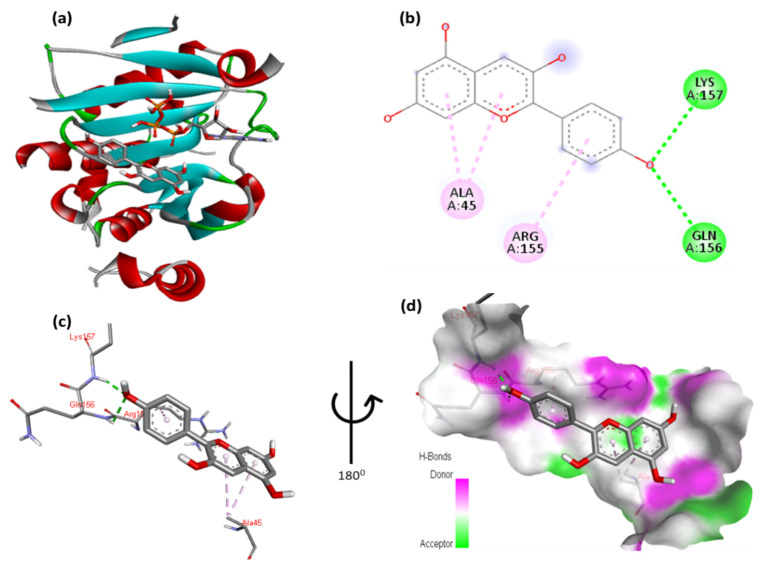
(**a**) Molecular docking analysis showing pelargonidin to the active site EIF4E protein. (**b**) Key interaction and visualization between protein residues with ligand in different colored dotted lines. A close-up view of the residues of the EIF4E protein interacting with the ligand is shown in (**c**,**d**), highlighting key binding interactions of the molecular level.

The docking results for pelargonidin with EIF4E are shown in Figure 4 and Table S5. The phenolic moiety of pelargonidin formed two hydrogen bonds with Gln156 and Lys157 and one hydrophobic interaction with Arg155. In addition, the double aromatic ring of the molecule possesses hydrophobic bonds with Ala45. Figure 4c,d show close-up views of the residues interacting with a molecule.

**Figure 5:**
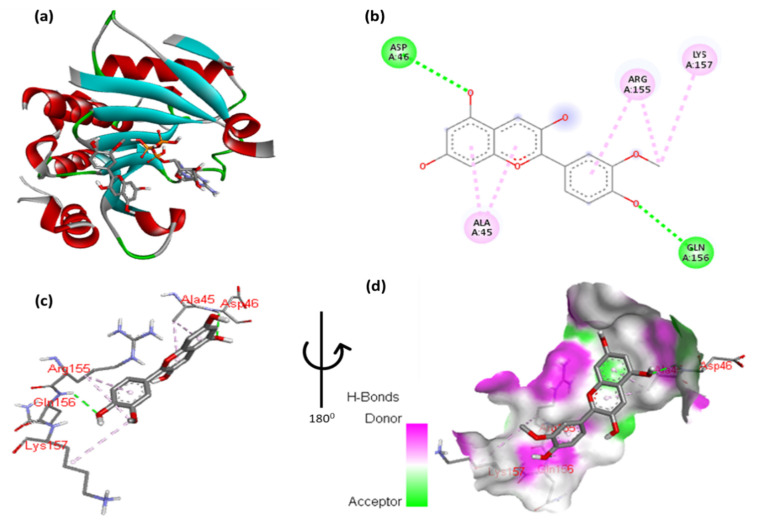
(**a**) Molecular docking analysis showing peonidin to the active site of the EIF4E protein. (**b**) Key interaction and visualization between peonidin and active site of EIF4E residues. A close-up view of interacting residue of EIF4E protein with ligand in (**c**,**d**), highlighting key binding interactions at the molecular level.

Figure 5 and Table S6 display the docking results of peonidin with EIF4E. The phenolic moiety of peonidin formed one hydrogen bond with Gln156, and two hydrogen interactions were established with the methoxy and aromatic moieties. The double aromatic ring of the molecule forms hydrogen bonds with Asp46 and hydrophobic interactions with Ala45. Figure 5c,d depict close-up views of the residues interacting with the molecule.

**Figure 6:**
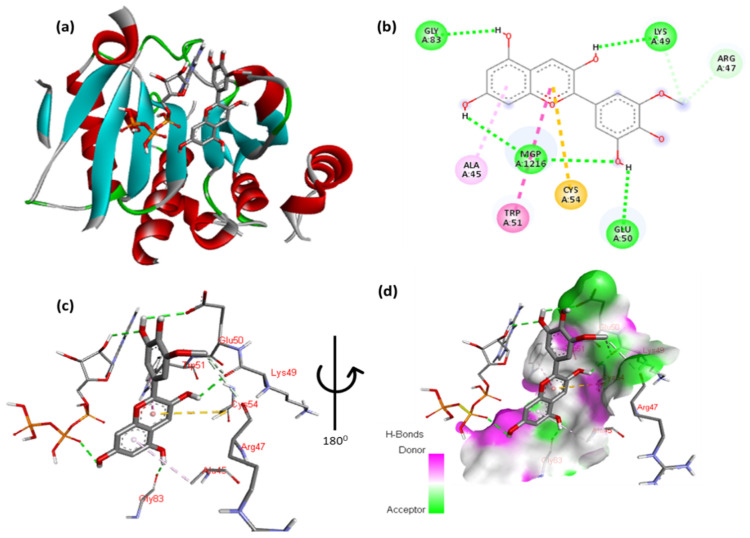
(**a**) Molecular docking analysis showing petunidin to the active sites of EIF4E protein. (**b**) Key interaction and visualization between ligand atoms and active site residues. A close-up view (**c**,**d**) of specific amino acid residues in the EIF4E protein interactions at the molecular level.

The docking results for petunidin with EIF4E are shown in Figure 6 and Table S7. The petunidin moiety, methoxyphenol, forms hydrogen bonds with Arg47 and Lys49. Additionally, Ag155 displayed one arene–arene interaction with the aromatic ring of the 2,6-dimethoxyphenol moiety and one hydrophobic interaction of the methoxy moiety with Lys157. Additionally, the double aromatic ring of the molecule possesses hydrophobic interactions with Trp51 and Ala45, whereas the hydroxy moiety displays hydrogen bonds with Lys49 and Gly83. Figure 6c,d show close-up views of the residues interacting with the molecule.

**Figure 7:**
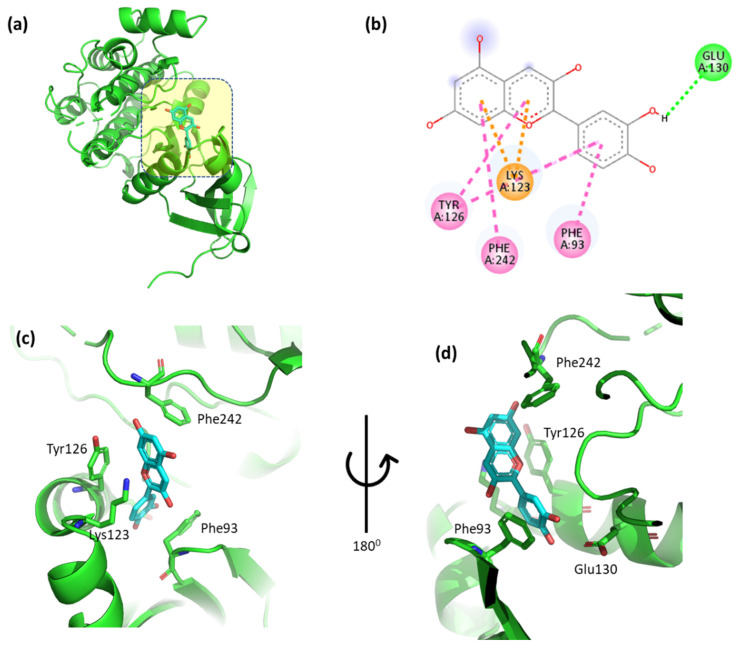
(**a**) Molecular docking analysis showing cyanidin to the active site of the Pdk1 protein. (**b**) Key in-teractions and visualization between the ligand and active site residues. A close-up views (**c**,**d**) highlight specific amino acid residues of the Pdk1 protein involved in binding, emphasizing the detailed interactions between the ligand and the protein.

The docking results for cyanidin with phosphoinositide-dependent kinase-1 (Pdk1) are shown in Figure 7 and Table S8. The 2,6-dimethoxyphenol moiety of cyanidin formed one hydrogen bond with Glu130 and one arene contacted Phe93. One arene–arene interaction was observed between Lys123 and a single and double aromatic ring in the molecule. In addition, the double aromatic ring of the molecule had hydrophobic connections with both Phe242 and Tyr126.

**Figure 8:**
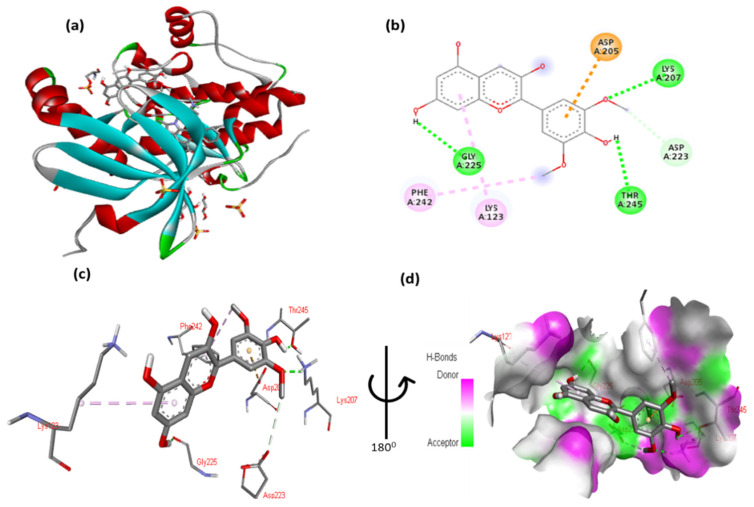
(**a**) Molecular docking analysis showing malvidin to the active site of Pdk1 protein. (**b**) Key inter-action and visualization between malvidin and Pdk1 active site residues, colored dotted lines rep-resenting various types of bonds. A close-up view (**c**,**d**) highlights specific amino acid residues of Pdk1 protein involved in binding, emphasizing the detailed interactions between the and protein.

The docking results for malvidin with Pdk1 are shown in Figure 8 and Table S9. The 2,6-dimethoxyphenol moiety of malvidin forms one hydrogen bond with Glu130 and one arene in contact with Phe93. One arene–arene interaction can be seen between Lys123 with a single and double aromatic ring in the molecule. In addition, the molecule’s double aromatic ring has hydrophobic connections with both Phe242 and Tyr126.

**Figure 9:**
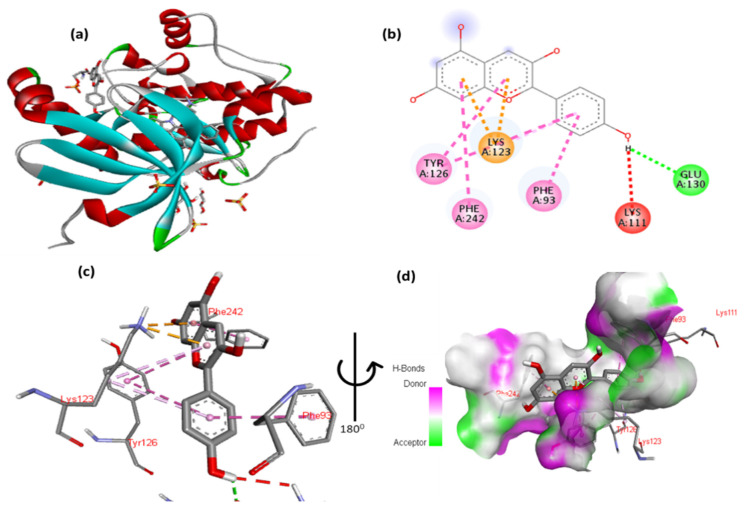
(**a**) Molecular docking analysis showing pelargonidin to the active site of Pdk1 protein. (**b**) Key interaction and visualization between pelargonidin and Pdk1 active site residues. A close-up view of Pdk1 protein interaction residue with ligands in (**c**,**d**), highlighting key binding interactions at the molecular level.

The docking results for pelargonidin with Pdk1 are shown in Figure 9 and Table S10. The 2,6-dimethoxyphenol moiety of pelargonidin forms one hydrogen bond with Glu130 and one arene in contact with Phe93. One arene–arene interaction can be seen between Lys123 with a single and double aromatic ring in the molecule. The double aromatic ring of the molecule had hydrophobic connections with Phe242 and Tyr126.

**Figure 10:**
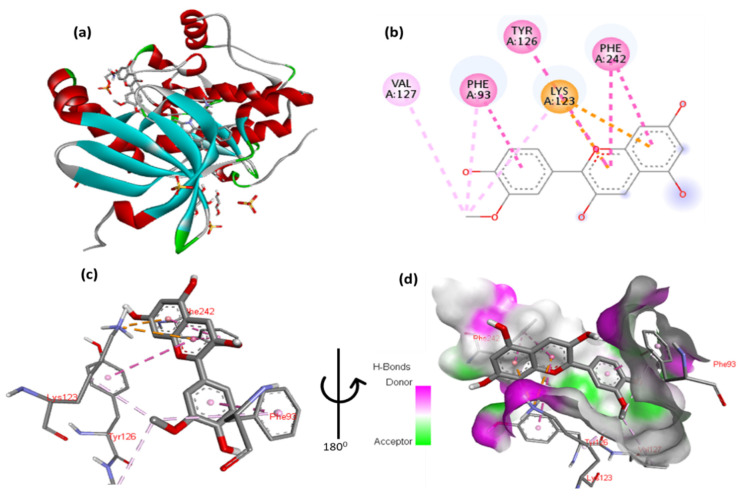
(**a**) Molecular docking analysis showing the binding of peonidin to the active site of Pdk1 protein. (**b**) Key interaction and visualization between peonidin and Pdk1 active site residues. A close-up view of Pdk1 protein interaction residue with ligands in (**c**,**d**), highlighting key binding interactions at the molecular level.

The docking results for peonidin with Pdk1 are shown in Figure 10 and Table S11. The 2,6-dimethoxyphenol moiety of peonidin formed one hydrogen bond with Glu130 and one arene contacted Phe93. One arene–arene interaction can be seen between Lys123 with a single and double aromatic ring in the molecule. The molecule’s double aromatic ring has hydrophobic connections with Phe242 and Tyr126.

**Figure 11:**
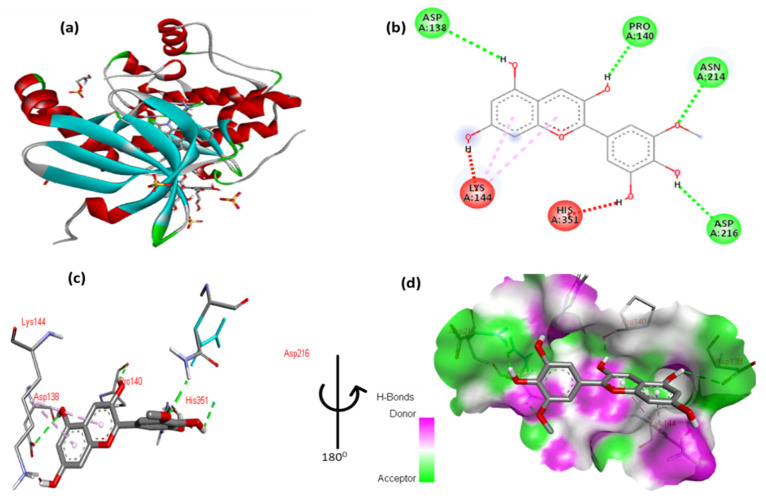
(**a**) Molecular docking analysis showing petunidin to the active site of Pdk1 protein. (**b**) Key inter-action and visualization of between petunidin and Pdk1 active site residues. A close-up of a Pdk1 protein interaction residue with ligands in (**c**,**d**), highlighting key binding interactions at the mo-lecular level.

The docking results of petunidin with Pdk1 are shown in Figure 11 and Table S12. The 2,6-dimethoxyphenol moiety of petunidin forms a hydrogen bond with Glu130 and one arene in contact with Phe93. One arene–arene interaction can be seen between Lys123 with a single and double aromatic ring in the molecule. In addition, the double aromatic ring of the molecule had hydrophobic connections with both Phe242 and Tyr126.

**Figure 12:**
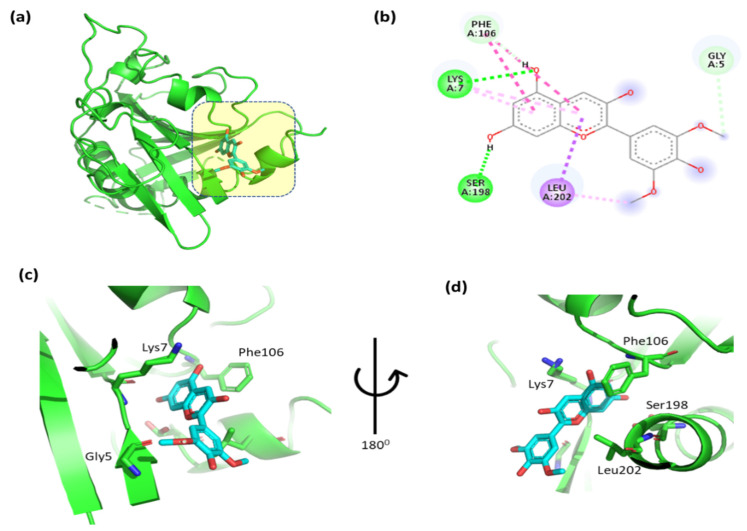
(**a**) Molecular docking analysis confirming the binding of cyanidin to the active site of Tsc2 protein. (**b**) Key interaction and visualization between cyanidin and EIF4E active site residues. A close-up view of Tsc2 protein residues interaction with the ligand in (**c**,**d**), highlighting key binding interactions at the molecular level.

The docking results of cyanidin with tuberous sclerosis-2 (Tsc2) are shown in Figure 12 and Table S13. The 2,6-dimethoxyphenol moiety of cyanidin forms one hydrophobic interaction with Gly5 and one hydrophobic contact with Leu202. Two hydrogen interactions were established between ser198 and Lys7 with a double aromatic ring in the molecule. In addition, the double aromatic ring of the molecules contains arene–arene with both Phe106.

**Figure 13:**
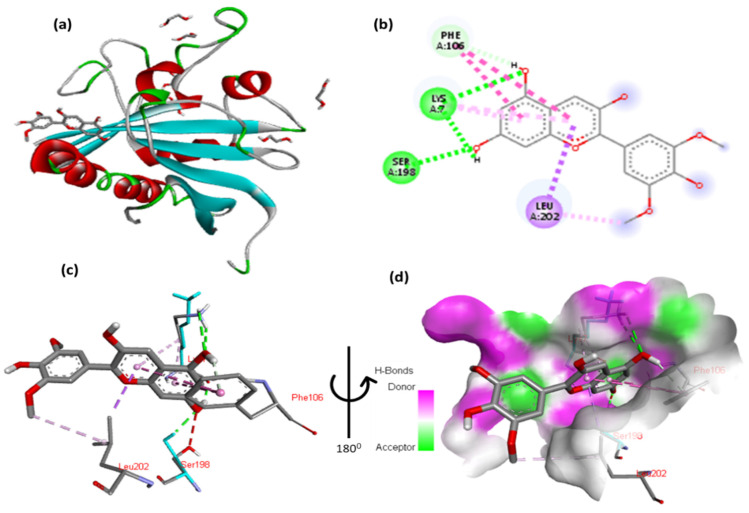
(**a**) Molecular docking analysis showing malvidin in the Tsc2 protein active site. (**b**) Key interaction and visualization between malvidin and Tsc2 active site residues. A close-up of Tsc2 protein in-teraction residue with the ligand in (**c**,**d**), highlighting key binding interactions at the molecular level.

The docking results for malvidin with Tsc2 are shown in Figure 13 and Table S14. The 2,6-dimethoxyphenol moiety of malvidin forms a hydrophobic interaction with Gly5 and one hydrophobic contact with Leu202. Two hydrogen interactions were established between ser198 and Lys7 with a double aromatic ring in the molecule. In addition, the double aromatic ring of the molecule contains arene–arene with both Phe106.

**Figure 14:**
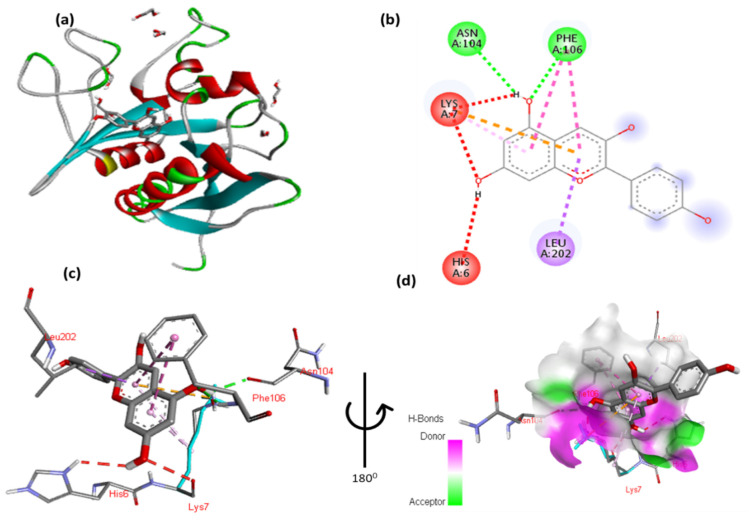
(**a**) Molecular docking analysis showing between pelargonidin to the Tsc2 protein active site. (**b**) Key interaction and visualization between pelargonidin and Tsc2 active site residues. A close-up view of Tsc2 protein interaction residue with ligand in (**c**,**d**), highlighting key binding interactions at the molecular level.

The docking results for pelargonidin with Tsc2 are shown in Figure 14 and Table S15. The 2,6-dimethoxyphenol moiety of pelargonidin formed a hydrophobic interaction with Gly5 and a hydrophobic contact with Leu202. Two hydrogen interactions were established between ser198 and Lys7, with a double aromatic ring in the molecule. In addition, the molecule’s double aromatic ring has arene–arene with both Phe106.

**Figure 15:**
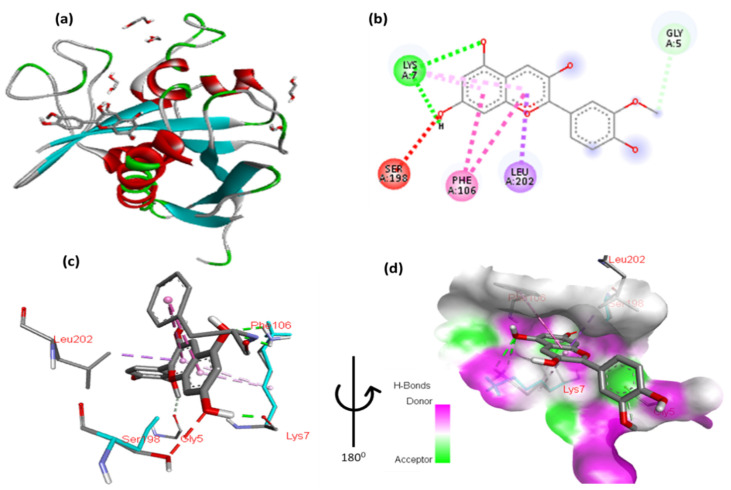
(**a**) Molecular docking analysis showing between peonidin to the active site of Tsc2 protein. (**b**) Key interaction and visualization between peonidin and Tcs2 active site residues. A close-up of Tsc2 protein interaction residue with ligand in (**c**,**d**), highlighting key binding interactions at the molecular level.

The docking results for peonidin with Tsc2 are shown in Figure 15 and Table S16. The 2,6-dimethoxyphenol moiety of peonidin formed a hydrophobic interaction with Gly5 and a hydrophobic contact with Leu202. Two hydrogen interactions were established between ser198 and Lys7 with a double aromatic ring in the molecule. In addition, the molecule’s double aromatic ring has arene–arene with both Phe106.

**Figure 16:**
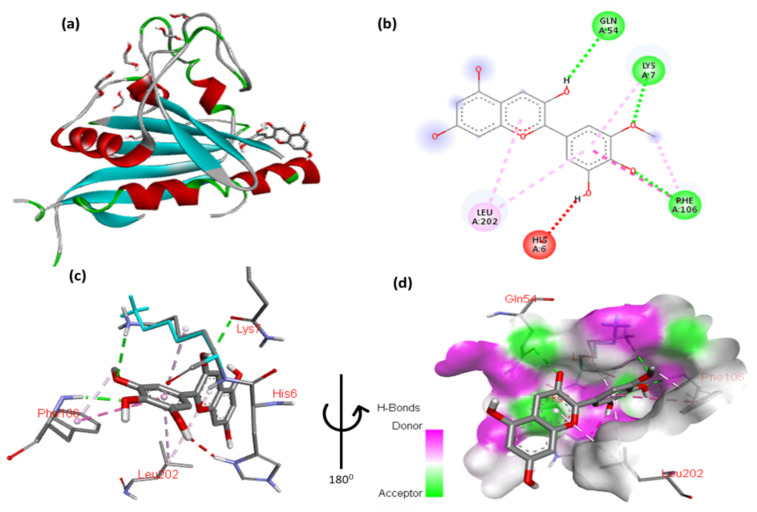
(**a**) Molecular docking analysis showing between petunidin to the active site of Tsc2 protein. (**b**) Key interaction and visualization between petunidin and Tsc2 active site residues. A close-up of the protein interaction residue with the ligand in (**c**,**d**), highlighting key binding interactions at the molecular level.

The docking results for petunidin with Tsc2 are shown in Figure 16 and Table S17. The 2,6-dimethoxyphenol moiety of petunidin formed a hydrophobic interaction with Gly5 and a hydrophobic contact with Leu202. Two hydrogen interactions were established between ser198 and Lys7 with a double aromatic ring in the molecule. In addition, the molecule’s double aromatic ring has arene–arene with both Phe106.

### 3.6. RT-qPCR Analysis of Gene Expression

**Figure 17:**
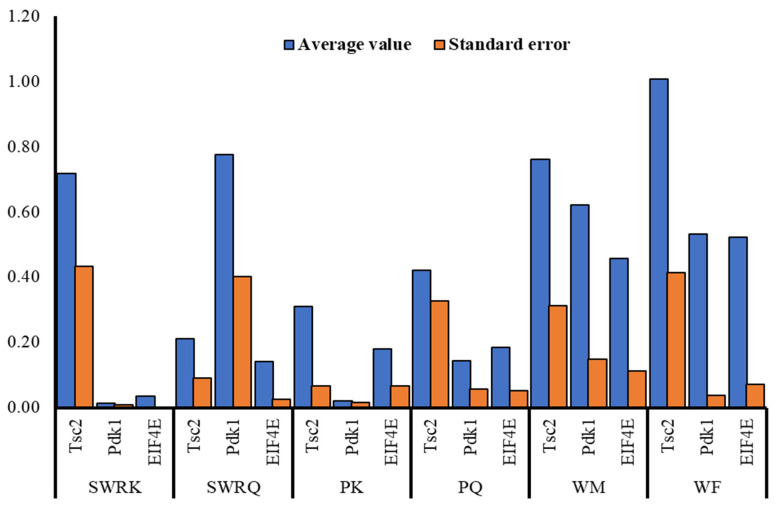
Average gene expression levels of Tsc2, Pdk1, and EIF4E across *Reticulitermes chinensis* castes with standard errors.

The RT-qPCR analysis reveals differential expression patterns of Tsc2, Pdk1, and EIF4E across various castes of *R. chinensis*. Tsc2 exhibits the highest expression levels in the WF (1.01 ± 0.41) and SWRK (0.72 ± 0.43) castes, while SWRQ shows the lowest expression (0.21 ± 0.09), suggesting caste-specific colors for Tsc2. Pdk1 expression is significantly elevated in SWRQ (0.78 ± 0.40), WM (0.62 ± 0.15), and WF (0.53 ± 0.04), whereas SWRK and PK exhibit minimal expression levels (0.01 ± 0.01 and 0.02 ± 0.01, respectively), indicating its potential functional importance in certain castes. EIF4E demonstrates peak expression in WF (0.52 ± 0.07) and WM (0.46 ± 0.11), with comparatively lower levels in SWRK (0.03 ± 0.00) and PK (0.18 ± 0.07), possibly reflecting increased protein synthesis requirements in worker castes (Figure 17).

Insulin resistance is a hallmark of an impaired insulin/insulin-like growth factor signaling (IIS) pathway, and anthocyanins have been shown to reduce insulin resistance [69]. Anthocyanins compounds enhance glucose uptake by activating AMP-activated protein kinase (AMPK) and inhibiting protein tyrosine phosphatase 1B (PTP1B) [70], a negative regulator of the IIS pathway, thereby promoting its activation [71,72]. Anthocyanins offer a range of health benefits, including antioxidant, anti-inflammatory, and insulin-resistance-reducing effects, which have made them a subject of interest in molecular docking studies due to their potential therapeutic properties [73,74,75]. In this context, the molecular docking of anthocyanin-related molecules (cyanidin, malvidin, pelargonidin, peonidin, and petunidin) with Pdk1, Tsc2, and EIF4E was conducted to assess the anti-aging potential of these compounds in termites.

The absorption, distribution, metabolism, elimination, and transport (ADMET) properties are critical for maintaining lead-like qualities and molecular stability. Several factors can influence ADMET properties, including the molecular size, octanol/water partition coefficient (calculated log P), H-bond acceptors, and H-bond donors [76]. In the purification process, delphinidin was found to violate one of the Lipinski’s rule of five, specifically the criterion regarding the H-bond donor, as it contains more than five such donors. The presence of more than five H-bond donors may affect molecular absorption, particularly via passive transport mechanisms [77]. Some studies suggest that the receptor–ligand interaction is influenced by protein folding, which is regulated by the balance between H-bond donor–acceptor interactions [78]. Additionally, the binding affinity of a ligand for its receptor can be modulated by the presence of water molecules, which interfere with the binding process and alter the strength of the interaction [79].

A compound’s physicochemical characteristics can provide insight into its ADMET properties but cannot reliably predict its affinity for a specific protein [80]. The docking score indicates the strength of the interaction between the ligand and its receptor [40,81], with a more negative docking score reflecting a higher binding affinity. The binding affinity of each anthocyanin to its target protein varies, and visualizing these interactions with different proteins has helped researchers gain a deeper understanding of the differences in binding affinities among these compounds.

In the MAPK pathway, delphinidin, pelargonidin, and peonidin exhibited the strongest binding properties among the anthocyanins, although they did not surpass the binding efficacy of known inhibiting ligands [40]. When compared with anthocyanins, inhibitors of MAPKAPK2 and MAPK10 showed a similar number of hydrophobic interactions and relatively high levels of H-bonding, as revealed by the interaction visualization results. The ability to form non-covalent interactions is directly correlated with the binding affinity of known inhibitors to their proteins; a higher capacity for such interactions typically corresponds to the strongest binding affinity [82]. The binding affinities of delphinidin with IGF1R and those of cyanidin and pelargonidin with Pdk1 in the IIS pathway were comparable, likely due to an equivalent number of non-covalent interactions. In contrast, malvidin and petunidin exhibited stronger binding affinities for Akt1, potentially due to greater number of hydrophobic interactions and H-bonds [82].

In general, anthocyanin compounds exhibit a high binding affinity for protein associated with AMPK, likely due to the significant number of hydrophobic interactions and H-bonds formed with sirt3, sirt6, and AMPK2. Among these, anthocyanin have a particularly strong propensity to binding with sirt6 [40]. Peonidin, which has a considerably lower number of hydroxy groups compared to other anthocyanins, ranks highest in the MAPK pathway but not in the insulin signaling pathway. This finding is noteworthy, as the top-binding anthocyanins vary for each protein involved in the insulin cascade [40]. Notably, malvidin holds the top position in binding to Akt-1, while securing second place for all other proteins. The substructures of the anthocyanins involved in non-covalent interactions differ for each protein, which may account for the variation in binding preferences. Anthocyanins share a common C6 substructure, responsible for hydrophobic interactions with MAPK proteins. For AMPK proteins, this substructure is denoted as C6, while, for insulin-signaling protein, it is referred to as C4. Due to the high number of carbon atoms in these substructures, hydrophobic interactions with protein are more likely to occur, potentially resulting in thermodynamic effects [83,84].

The substructures R5 and R3 are involved in the formation of H-bonds with MAPK, while R3′ and R5 interact with AMPK, and R3, R5, and R5′ are implicated in interactions with insulin signaling proteins. There is a possibility that the substructures R5, R3, R5′, and R3′ each have a unique function, such as acting as H-bond donors, acceptors, or both simultaneously. The hydroxyl groups of anthocyanin may influence the H-bond between the protein and anthocyanin [40]. These findings suggest that different anthocyanin compounds may exhibit varying binding affinities for proteins associated with the aging process, likely due to differences in their substructures. These variations may affect the non-covalent binding interactions and binding affinities of the compounds.

## 4. Conclusions

This study highlights the significant therapeutic potential of anthocyanins in age-related metabolic research in social insects. Through a comprehensive molecular docking analysis, we have elucidated the binding affinities and interactions of various anthocyanins with key proteins involved in the insulin/insulin-like growth factor and IIS pathway. Our findings reveal that specific anthocyanin compounds exhibit optimal binding results, forming hydrogen bonds, hydrophobic interactions, and arene–arene interactions with target proteins. These molecular interactions suggest a potential mechanism by which anthocyanins may modulate the IIS pathway, potentially influencing insulin sensitivity and metabolic regulation in social insects. These results emphasize the need for further investigation into the biological mechanisms underlying these interactions, as understanding their impact on cellular pathways could lead to targeted therapeutic strategies for managing conditions such as insulin resistance and other age-related diseases. Additionally, this research opens new avenues for incorporating anthocyanin-rich foods into dietary guidelines, offering a natural approach to improving health outcomes. Challenges to this study may include the limitations of the in silico molecular docking analysis, which may not fully represent the complex biological environment in living organisms. Additionally, translating these computational findings to real-world applications and validating the results through in vivo studies could be challenging. To validate these in silico findings and assess the therapeutic potential of anthocyanins in clinical settings, future research could also investigate the synergistic effects of different anthocyanins or their combinations with other phytochemicals. To advance this research, both animal models and human subjects could be used for in vivo studies. Animal models, such as rodents or social insects mentioned in the study, could provide initial insights into the physiological effects of anthocyanins on insulin sensitivity and metabolic parameters could examine changes in gene expression, protein levels, and metabolic markers in response to anthocyanin supplementation. Following promising results in animal models, human clinical trials could be conducted to assess the efficacy and safety of anthocyanin interventions in managing insulin resistance and age-related metabolic diseases. These trials could involve long-term dietary interventions with anthocyanin-rich foods or supplements, monitoring various health markers, and evaluating the impact on insulin sensitivity and overall metabolic health in different age groups and populations. This research contributes to the growing body of evidence supporting the health benefits of phytochemicals, particularly anthocyanins and their potential applications in modern medicine and preventive healthcare strategies.

## Figures and Tables

**Table 1:** The three selected genes from *R. chinensis*.

Gene ID	Symbol	Primers Sequences
	Beta-actin	Forward: CCCAACACAGCGTCTTACAA Reverse: CAGATGTCCTCAGCTTCACG
Unigene 0082575	*PdK1*	Forward: TCCTCCTCCTGCTACTGCTGAAG Reverse: CGACATATGACGGAGTAGGTGGTG
Unigene 0092210	*Tsc2*	Forward: AGTGGTGCTAACATGCCTGC Reverse: ACCTTCCAGCTGCTCTGACA
Unigene 0011832	*EIF4E*	Forward: GGATCTCGTCTTGGCCGTCATTG Reverse: AGCAACCTCGTGAGCCACTCC

**Table 2:** Biochemical and structural analysis of Pdk1, Tsc2, and EIF4E proteins.

	Pdk1	Tsc2	EIF4E
Number of amino acids	290	65	296
Molecular weight	32,997.1	7229.54	33,978.5
Theoretical pI	9.58	10.77	8.93
Negatively charged residues (Asp + Glu)	6	3	16
Positively charged residues (Arg + Lys)	24	14	32
Carbon (C)	1519	313	1541
Hydrogen (H)	2354	543	2422
Nitrogen (N)	404	99	400
Oxygen (O)	378	90	402
Sulfur (S)	21	3	31
Total number of atoms	4676	1048	4796
Ext. coefficient	76,150	1615	40,935
Abs 0.1% (=1 g/L) (Cys residues form cystines)	2.308	0.223	1.205
Ext. coefficient	75,400	1490	39,310
Abs 0.1% (=1 g/L) (Cys residues are reduced)	2.285	0.206	1.157
Instability index (II)	29.21	59.09	39.26
Aliphatic index	105.14	91.54	106.25
GRAVY	0.292	−0.309	0.307

**Table 3:** Comparative analysis of Pdk1, Tsc2, and EIF4E proteins.

		Pdk1	Tsc2	EIF4E
Metrics	Hydrophobic Fitness	−17	−15	−18
	Isoelectric Point	8	8	5
	Number of Residues	275	191	211
	Mass (Da)	31,845	21,894	24,684
	Mean Packing Density	58	57	58
BUDE force field results	Total Energy	−4272	-	-
	Steric	251	-	-
	Desolvation	−2873	-	-
	Charge	−1650	-	-
EvoEF2 energy function results-summary	Total Energy	−1227	−934	−1082
	Reference	−54	−40	−32
	Intra-Residue	598	391	435
	Inter-Residue—Same Chain	−1771	−1285	−1380
	Inter-Residue—Different Chains	0	0	−105
DFIRE2 energy function results	Total Energy	−523	−343	−404
Rosetta energy function results	Total Energy	81	−175	−276
	Reference	72	72	66
	VDW Attractive	−1697	−1100	−1259
	VDW Repulsive	366	76	146
	VDW Repulsive Intra-Residue	5	2	2
	Electrostatics	−383	−239	−332
	Solvation Isotropic	995	670	752
	Solvation Anisotropic Polar Atoms	−34	−23	−32
	Solvation Isotropic Intra Residue	62	38	53
	HB Long Range Backbone	−46	−51	−64
	HB Short Range Backbone	−85	−46	−63
	HB Backbone Sidechain	−33	−40	−21
	HB Sidechain Sidechain	−14	−21	−22
	Disulfide Bridges	0	0	0
	Backbone Torsion Preference	35	9	18
	Amino Acid Propensity	−29	−31	−26
	Dunbrack Rotamer	781	381	434
	Omega Penalty	60	89	62
	Open Proline Penalty	25	40	11
	Tyrosine χ3 Dihedral Angle Penalty	0	0	0
Aggregation propensity results	Total Score	−227	−103	−181
	Average Score	−0.83	−0.54	−0.86
	Minimum Score	−4.31	−4.31	−4.48
	Maximum Score	1.9	2.9	1.56

## Data Availability

All data generated or analyzed during this study are included in this published article (and its Supplementary Information Files).

## Supplementary Materials

The following supporting information can be downloaded at: https://www.mdpi.com/article/10.3390/cimb47020087/s1.
