# Supplementary material for: Anthocyanin-Binding Affinity and Non-Covalent Interactions with IIS-Pathway-Related Protein Through Molecular Docking
Source: Curr Issues Mol Biol. 2025 Jan 29;47(2):87. doi: 10.3390/cimb47020087 (PMC11854422; doi:10.3390/cimb47020087)
Supplement: Supplementary file 1 [file cimb-47-00087-s001.zip › cimb-3448783-supplementary.pdf]

Supplementary list of Tables

Table S1. Comparing the lead-likeness of anthocyanin and other known ligands with the use of the Lipinski Rule of five

Compounds	Mass	Log P	H bond acceptor	H bond donor	Ro5 violation
Reference	≤500 g/mol	≤5	≤10	≤5	≤1
Cyanidin	287.24	2.9089	6	5	0
Delphinidin	338.69	-0.3815	7	6*	1
Malvidin	331.29	3.2205	7	4	0
Pelargonidin	271.24	3.2033	5	4	0
Peonidin	301.27	3.2119	6	4	0
Petunidin	317.26	2.9175	7	5	0

Table S2. Ranking of the binding energies of the ligands found in the several genes associated with the ageing process

MAPK Pathway			AMPK-related Pathway			Insulin Signalling Pathway		
MAPKAP2	MAPK10	BRAF	SIRT3	SIRT6	AMPK2	IGF1R	PDK-1	Akt1
PDY<br>-7.3	ANP<br>-9.0	215<br>-10.5	Delphinidin<br>-9.3	AR6<br>-11.3	Peonidin<br>-9.3	ANO<br>-7.3	STU<br>-8.8	Malvidin<br>-8.3
Delphinidin<br>-7.1	Pelargonidin<br>-8.3	Peonidin<br>-8.8	Delphinidin<br>-9.5	Petunidin<br>-9.6	Cyanidin<br>-9.2	Delphinidin<br>-7.2	Cyanidin<br>-8.7	Petunidin<br>-8.3
Cyanidin<br>-6.9	Peonidin<br>-8.3	Cyanidin<br>-9.3	Peonidin<br>-8.8	Delphinidin<br>-9.5	TAK<br>-9.2	Petunidin<br>-7.0	Pelargonidin<br>-8.7	Delphinidin<br>-8.0
Malvidin<br>-6.9	Delphinidin<br>-8.2	Malvidin<br>-9.3	Petunidin<br>-8.8	Cyanidin<br>-9.3	Pelargonidin<br>-9.2	Malvidin<br>-6.9	Petunidin<br>-8.6	Cyanidin<br>-7.9
Petunidin<br>-6.8	Malvidin<br>-8.2	Pelargonidin<br>-9.3	AR6<br>-8.6	Pelargonidin<br>-9.3	Petunidin<br>-9.0	Cyanidin<br>-6.9	Delphinidin<br>-8.5	Peonidin<br>-7.9
Pelargonidin<br>-6.8	Petunidin<br>-8.2	Petunidin<br>-9.3	Cyanidin<br>-7.3	Peonidin<br>-9.3	Delphinidin<br>-8.8	Pelargonidin<br>-6.8	Peonidin<br>-8.4	Pelargonidin<br>-7.6
Peonidin<br>-6.8	Cyanidin<br>-8.0	Delphinidin<br>-9.2	Malvidin<br>-5.5	Malvidin<br>-89.2	Malvidin<br>-8.8	Peonidin<br>-6.8	Malvidin<br>-8.2	XFE<br>-7/1

Table S3: Types of interactions in the reference ligand cyanidin with EIF4E receptor.

Name of amino acid	Distance	Nature of bond	Type of interactions
A:ASP46:HN - :UNK0:O	2.72341	Hydrogen Bond	Conventional Hydrogen Bond
A:GLN156:HN - :UNK0:O	2.18772	Hydrogen Bond	Conventional Hydrogen Bond
:UNK0:C - A:MGP1216:O1C	3.55589	Hydrogen Bond	Carbon Hydrogen Bond
:UNK0:C - A:ARG155	4.05965	Hydrophobic	Alkyl
:UNK0:C - A:LYS157	4.56434	Hydrophobic	Alkyl

A:TRP82 - :UNK0:C	5.0004	Hydrophobic	Pi-Alkyl
:UNK0 - A:ALA45	4.26792	Hydrophobic	Pi-Alkyl
:UNK0 - A:ALA45	4.11728	Hydrophobic	Pi-Alkyl
:UNK0 - A:ARG155	4.32515	Hydrophobic	Pi-Alkyl

**Table S4:** Types of interactions in the reference ligand malvidin with EIF4E receptor.			
**Name of amino acid**	**Distance**	**Nature of bond**	**Type of interactions**
A:ASP46:HN - :UNK0:O	2.72418	Hydrogen Bond	Conventional Hydrogen Bond
A:GLN156:HN - :UNK0:O	2.18763	Hydrogen Bond	Conventional Hydrogen Bond
:UNK0:C - A:MGP1216:O1C	3.54886	Hydrogen Bond	Carbon Hydrogen Bond
:UNK0:C - A:ARG155	4.05572	Hydrophobic	Alkyl
:UNK0:C - A:LYS157	4.57419	Hydrophobic	Alkyl
A:TRP82 - :UNK0:C	4.99986	Hydrophobic	Pi-Alkyl
:UNK0 - A:ALA45	4.26756	Hydrophobic	Pi-Alkyl
:UNK0 - A:ALA45	4.11575	Hydrophobic	Pi-Alkyl
:UNK0 - A:ARG155	4.324	Hydrophobic	Pi-Alkyl

**Table S5:** Types of interactions in the reference ligand pelargonidin with EIF4E receptor.			
**Name of amino acid**	**Distance**	**Nature of bond**	**Type of interactions**
A:GLN156:HN - :UNK0:O	2.60877	Hydrogen Bond	Conventional Hydrogen Bond
A:LYS157:HN - :UNK0:O	2.32748	Hydrogen Bond	Conventional Hydrogen Bond
:UNK0 - A:ALA45	4.2785	Hydrophobic	Pi-Alkyl
:UNK0 - A:ALA45	4.09383	Hydrophobic	Pi-Alkyl
:UNK0 - A:ARG155	4.32856	Hydrophobic	Pi-Alkyl

**Table S6:** Types of interactions in the reference ligand peonidin with EIF4E receptor.			
**Name of amino acid**	**Distance**	**Nature of bond**	**Type of interactions**
A:ASP46:HN - :UNK0:O	2.75509	Hydrogen Bond	Conventional Hydrogen Bond
A:GLN156:HN - :UNK0:O	2.58593	Hydrogen Bond	Conventional Hydrogen Bond
:UNK0:C - A:ARG155	4.32591	Hydrophobic	Alkyl
:UNK0:C - A:LYS157	4.49067	Hydrophobic	Alkyl
:UNK0 - A:ALA45	4.28311	Hydrophobic	Pi-Alkyl
:UNK0 - A:ALA45	4.08753	Hydrophobic	Pi-Alkyl
:UNK0 - A:ARG155	4.28197	Hydrophobic	Pi-Alkyl

**Table S7:** Types of interactions in the reference ligand peonidin with EIF4E receptor.			
**Name of amino acid**	**Distance**	**Nature of bond**	**Type of interactions**
A:MGP1216:HO2' - :UNK0:O	2.70464	Hydrogen Bond	Conventional Hydrogen Bond
:UNK0:H - A:GLU50:OE1	2.83007	Hydrogen Bond	Conventional Hydrogen Bond
:UNK0:H - A:LYS49:O	2.41858	Hydrogen Bond	Conventional Hydrogen Bond
:UNK0:H - A:GLY83:O	2.0662	Hydrogen Bond	Conventional Hydrogen Bond

:UNK0:H - A:MGP1216:O2B	2.595	Hydrogen Bond	Conventional Hydrogen Bond
:UNK0:C - A:ARG47:O	3.69146	Hydrogen Bond	Carbon Hydrogen Bond
:UNK0:C - A:LYS49:O	3.78482	Hydrogen Bond	Carbon Hydrogen Bond
A:CYS54:SG - :UNK0	5.65491	Other	Pi-Sulfur
A:TRP51 - :UNK0	5.6925	Hydrophobic	Pi-Pi T-shaped
:UNK0 - A:ALA45	5.16308	Hydrophobic	Pi-Alkyl

Table S8: Types of interactions in the reference ligand cyanidin with Pdk1 receptor.

**Name of amino acid**	**Distance**	**Nature of bond**	**Type of interactions**
:UNK0:H - A:GLU130:OE1	2.1375	Hydrogen Bond	Conventional Hydrogen Bond
A:LYS123:NZ - :UNK0	3.42936	Electrostatic	Pi-Cation
A:LYS123:NZ - :UNK0	3.60019	Electrostatic	Pi-Cation
A:PHE93 - :UNK0	4.44317	Hydrophobic	Pi-Pi Stacked
A:TYR126 - :UNK0	5.83465	Hydrophobic	Pi-Pi Stacked
A:TYR126 - :UNK0	5.1212	Hydrophobic	Pi-Pi T-shaped
A:PHE242 - :UNK0	5.08468	Hydrophobic	Pi-Pi T-shaped
:UNK0 - A:LYS123	5.34461	Hydrophobic	Pi-Alkyl
:UNK0 - A:LYS123	4.88771	Hydrophobic	Pi-Alkyl

Table S9: Types of interactions in the reference ligand malvidin with Pdk1 receptor.

**Name of amino acid**	**Distance**	**Nature of bond**	**Type of interactions**
A:LYS207:HZ1 - :UNK0:O	2.16645	Hydrogen Bond	Conventional Hydrogen Bond
:UNK0:H - A:THR245:OG1	2.43151	Hydrogen Bond	Conventional Hydrogen Bond
:UNK0:H - A:GLY225:O	2.03902	Hydrogen Bond	Conventional Hydrogen Bond
:UNK0:C - A:ASP223:OD2	3.4647	Hydrogen Bond	Carbon Hydrogen Bond
A:ASP205:OD2 - :UNK0	3.81702	Electrostatic	Pi-Anion
A:PHE242 - :UNK0:C	4.72982	Hydrophobic	Pi-Alkyl
:UNK0 - A:LYS123	5.48829	Hydrophobic	Pi-Alkyl

Table S10: Types of interactions in the reference ligand pelargonidin with Pdk1 receptor.

**Name of amino acid**	**Distance**	**Nature of bond**	**Type of interactions**
:UNK0:H - A:GLU130:OE1	2.08737	Hydrogen Bond	Conventional Hydrogen Bond
A:LYS123:NZ - :UNK0	3.42235	Electrostatic	Pi-Cation
A:LYS123:NZ - :UNK0	3.60947	Electrostatic	Pi-Cation
A:PHE93 - :UNK0	4.43103	Hydrophobic	Pi-Pi Stacked
A:TYR126 - :UNK0	5.81116	Hydrophobic	Pi-Pi Stacked
A:TYR126 - :UNK0	5.14058	Hydrophobic	Pi-Pi T-shaped
A:PHE242 - :UNK0	5.09194	Hydrophobic	Pi-Pi T-shaped
:UNK0 - A:LYS123	5.38348	Hydrophobic	Pi-Alkyl
:UNK0 - A:LYS123	4.91212	Hydrophobic	Pi-Alkyl

**Table S11:** Types of interactions in the reference ligand peonidin with Pdk1 receptor			
**Name of amino acid**	**Distance**	**Nature of bond**	**Type of interactions**
A:LYS123:NZ - :UNK0	4.22854	Electrostatic	Pi-Cation
A:LYS123:NZ - :UNK0	3.98037	Electrostatic	Pi-Cation
A:PHE93 - :UNK0	3.89885	Hydrophobic	Pi-Pi Stacked
A:TYR126 - :UNK0	5.62125	Hydrophobic	Pi-Pi Stacked
A:PHE242 - :UNK0	5.09572	Hydrophobic	Pi-Pi T-shaped
A:PHE242 - :UNK0	4.85925	Hydrophobic	Pi-Pi T-shaped
:UNK0:C - A:LYS123	3.57937	Hydrophobic	Pi-Alkyl
:UNK0:C - A:VAL127	4.88823	Hydrophobic	Pi-Alkyl
A:PHE93 - :UNK0:C	5.46748	Hydrophobic	Conventional Hydrogen Bond

**Table S12:** Types of interactions in the reference ligand petunidin with Pdk1 receptor			
**Name of amino acid**	**Distance**	**Nature of bond**	**Type of interactions**
A:ASN214:HD22 - :UNK0:O	2.21446	Hydrogen Bond	Conventional Hydrogen Bond
:UNK0:H - A:ASP216:OD2:B	2.40702	Hydrogen Bond	Conventional Hydrogen Bond
:UNK0:H - A:PRO140:O	2.08464	Hydrogen Bond	Conventional Hydrogen Bond
:UNK0:H - A:ASP138:OD1	2.93257	Hydrogen Bond	Conventional Hydrogen Bond
:UNK0 - A:LYS144	5.17469	Hydrophobic	Pi-Alkyl
:UNK0 - A:LYS144	4.35452	Hydrophobic	Pi-Alkyl

**Table S13:** Types of interactions in the reference ligand cyanidin with Tsc2 receptor.			
**Name of amino acid**	**Distance**	**Nature of bond**	**Type of interactions**
A:LYS7:HZ1 - :UNK0:O	2.60782	Hydrogen Bond	Conventional Hydrogen Bond
A:LYS7:HZ3 - :UNK0:O	2.82082	Hydrogen Bond	Conventional Hydrogen Bond
:UNK0:H - A:SER198:OG	2.26465	Hydrogen Bond	Conventional Hydrogen Bond
:UNK0:C - A:GLY5:O	3.70185	Hydrogen Bond	Carbon Hydrogen Bond
:UNK0:H - A:PHE106	2.58669	Hydrogen Bond	Pi-Donor Hydrogen Bond
A:LEU202:CD2 - :UNK0	3.83055	Hydrophobic	Pi-Sigma
A:PHE106 - :UNK0	5.44086	Hydrophobic	Pi-Pi Stacked
A:PHE106 - :UNK0	4.71908	Hydrophobic	Pi-Pi Stacked
:UNK0:C - A:LEU202	4.05033	Hydrophobic	Alkyl
:UNK0 - A:LYS7	5.1032	Hydrophobic	Pi-Alkyl
:UNK0 - A:LYS7	4.4427	Hydrophobic	Pi-Alkyl

**Table S14:** Types of interactions in the reference ligand malvidin with Tsc2 receptor.			
**Name of amino acid**	**Distance**	**Nature of bond**	**Type of interactions**
A:LYS7:HZ1 - :UNK0:O	2.62094	Hydrogen Bond	Conventional Hydrogen Bond
A:LYS7:HZ3 - :UNK0:O	2.84127	Hydrogen Bond	Conventional Hydrogen Bond
A:SER198:HG:B - :UNK0:O	2.15232	Hydrogen Bond	Conventional Hydrogen Bond
:UNK0:H - A:LYS7:O	1.80396	Hydrogen Bond	Conventional Hydrogen Bond
:UNK0:H - A:PHE106	2.57481	Hydrogen Bond	Pi-Donor Hydrogen Bond

A:LEU202:CD2 - :UNK0	3.86554	Hydrophobic	Pi-Sigma
A:PHE106 - :UNK0	5.46949	Hydrophobic	Pi-Pi Stacked
A:PHE106 - :UNK0	4.7337	Hydrophobic	Pi-Pi Stacked
:UNK0:C - A:LEU202	4.65654	Hydrophobic	Alkyl
:UNK0 - A:LYS7	5.05793	Hydrophobic	Pi-Alkyl
:UNK0 - A:LYS7	4.43529	Hydrophobic	Pi-Alkyl

Table S15: Types of interactions in the reference ligand pelargonidin with Tsc2 receptor.

Name of amino acid	Distance	Nature of bond	Type of interactions
A:PHE106:HN - :UNK0:O	2.38035	Hydrogen Bond	Conventional Hydrogen Bond
:UNK0:H - A:ASN104:O	2.19461	Hydrogen Bond	Conventional Hydrogen Bond
A:LYS7:NZ:B - :UNK0	4.96021	Electrostatic	Pi-Cation
A:LEU202:CD2 - :UNK0	3.90554	Hydrophobic	Pi-Sigma
A:PHE106 - :UNK0	5.12249	Hydrophobic	Pi-Pi Stacked
A:PHE106 - :UNK0	4.75052	Hydrophobic	Pi-Pi Stacked
:UNK0 - A:LYS7	5.39187	Hydrophobic	Pi-Alkyl
:UNK0 - A:LYS7	4.35694	Hydrophobic	Pi-Alkyl

Table S16: Types of interactions in the reference ligand peonidin with Tsc2 receptor.

Name of amino acid	Distance	Nature of bond	Type of interactions
A:LYS7:HZ1 - :UNK0:O	2.63455	Hydrogen Bond	Conventional Hydrogen Bond
A:LYS7:HZ3 - :UNK0:O	2.85351	Hydrogen Bond	Conventional Hydrogen Bond
:UNK0:H - A:LYS7:O	1.82215	Hydrogen Bond	Conventional Hydrogen Bond
:UNK0:C - A:GLY5:O	3.74216	Hydrogen Bond	Carbon Hydrogen Bond
A:LEU202:CD2 - :UNK0	3.90567	Hydrophobic	Pi-Sigma
A:PHE106 - :UNK0	5.44283	Hydrophobic	Pi-Pi Stacked
A:PHE106 - :UNK0	4.70116	Hydrophobic	Pi-Pi Stacked
:UNK0 - A:LYS7	5.00086	Hydrophobic	Pi-Alkyl
:UNK0 - A:LYS7	4.43026	Hydrophobic	Pi-Alkyl

Table S17: Types of interactions in the reference ligand petunidin with Tsc2 receptor.

Name of amino acid	Distance	Nature of bond	Type of interactions
A:LYS7:HZ1 - :UNK0:O	2.86156	Hydrogen Bond	Conventional Hydrogen Bond
A:PHE106:HN - :UNK0:O	2.02657	Hydrogen Bond	Conventional Hydrogen Bond
:UNK0:H - A:GLN54:OE1	2.57734	Hydrogen Bond	Conventional Hydrogen Bond
A:PHE106 - :UNK0	5.15053	Hydrophobic	Pi-Pi Stacked
A:PHE106 - :UNK0:C	3.74152	Hydrophobic	Pi-Alkyl
:UNK0 - A:LEU202	5.39935	Hydrophobic	Pi-Alkyl
:UNK0 - A:LYS7	4.29653	Hydrophobic	Pi-Alkyl
:UNK0 - A:LEU202	5.40902	Hydrophobic	Pi-Alkyl
